# Screening of an endothelial cDNA library identifies the C-terminal region of Nedd5 as a novel autoantigen in systemic lupus erythematosus with psychiatric manifestations

**DOI:** 10.1186/ar1759

**Published:** 2005-05-20

**Authors:** Paola Margutti, Maurizio Sorice, Fabrizio Conti, Federica Delunardo, Mauro Racaniello, Cristiano Alessandri, Alessandra Siracusano, Rachele Riganò, Elisabetta Profumo, Guido Valesini, Elena Ortona

**Affiliations:** 1 Dipartimento di Malattie Infettive, Parassitarie e Immunomediate, Istituto Superiore di Sanità, Rome, Italy; 2 Dipartimento di Medicina Sperimentale e Patologia, Cattedra di Reumatologia, Università 'La Sapienza', Rome, Italy; 3 Dipartimento di Clinica e Terapia Medica Applicata, Cattedra di Reumatologia, Università 'La Sapienza', Rome, Italy

## Abstract

Anti-endothelial-cell antibodies are associated with psychiatric manifestations in systemic lupus erythematosus (SLE). Our primary aim in this study was to seek and characterize molecules that behave as endothelial autoantigens in SLE patients with psychiatric manifestations. By screening a cDNA library from human umbilical artery endothelial cells with serum from an SLE patient with psychosis, we identified one positive strongly reactive clone encoding the C-terminal region (C-ter) of Nedd5, an intracytoplasmatic protein of the septin family. To evaluate anti-Nedd5 serum immunoreactivity, we analyzed by ELISA specific IgG responses in 17 patients with SLE and psychiatric manifestations (group A), 34 patients with SLE without psychiatric manifestations (group B), 20 patients with systemic sclerosis, 20 patients with infectious mononucleosis, and 35 healthy subjects. IgG specific to Nedd5 C-ter was present in 14 (27%) of the 51 SLE patients. The mean optical density value for IgG immunoreactivity to Nedd5 C-ter was significantly higher in patients of group A than in those of group B, those with infectious mononucleosis, or healthy subjects (0.17 ± 0.14 vs, respectively, 0.11 ± 0.07, *P *= 0.04; 0.11 ± 0.06, *P *= 0.034; and 0.09 ± 0.045, *P *= 0.003, on Student's *t*-test). Moreover, IgG immunoreactivity to Nedd5 C-ter was significantly higher in patients with systemic sclerosis than in patients of group B or healthy subjects (0.18 ± 0.18 vs, respectively, 0.11 ± 0.07, *P *= 0.046; and 0.09 ± 0.045, *P *= 0.003). The percentage of patients with anti-Nedd5 C-ter serum IgG was higher in group A than in group B (8 (47%) of 17, vs 6 (17%) of 34, *P *= 0.045, on Fisher's exact test). In order to clarify a possible mechanism by which Nedd5 might be autoantigenic, we observed that Nedd5 relocated from cytoplasm to the plasma membrane of EAhy926 endothelial cells after apoptotic stimuli. In conclusion, Nedd5 is a novel autoantigen of potential clinical importance that could be successfully used for a more thorough investigation of the pathogenesis of psychiatric manifestations in SLE. Although anti-Nedd5 autoantibodies are not specific to SLE, they are significantly associated with neuropsychiatric SLE and may represent immunological markers of psychiatric manifestations in this pathology.

## Introduction

Symptoms originating from the central nervous system occur in 14 to 75% of patients with systemic lupus erythematosus (SLE) and are extremely diverse, including neurological and psychiatric syndromes [1]. In 1999, the American College of Rheumathology defined 19 distinct neuropsychiatric syndromes associated with SLE, including psychosis and depression [2,3]. Neuropsychiatric SLE remains an enigmatic manifestation in lupus. In fact, conflicting results have been reported to clarify associations between neuropsychiatric manifestations and serum antibodies against neuronal antigens, ribosomes, and phospholipids [4]. Recently, we demonstrated an association between the presence of anti-endothelial-cell antibodies (AECAs) and psychiatric manifestations, such as psychosis and depression in SLE, suggesting a possible mechanism underlying psychiatric symptoms [5]. By activating endothelial cells, AECAs up-regulate the expression of adhesion molecules as well as the secretion of cytokines and chemokines. Until recently, few published data have been available on the identity of endothelial cell autoantigens in immune disorders [6-11]. Identifying endothelial autoantigens involved in the autoimmune processes during neuropsychiatric SLE could help to explain the pathogenetic mechanisms involved in the initiation and progression of psychiatric symptoms.

Our primary aim in this study was to seek and characterize molecules that behave as endothelial autoantigens in neuropsychiatric SLE. By screening a cDNA library from human umbilical artery endothelial cells (HUAECs) with serum from an SLE patient with psychosis, we identified one strongly reactive clone encoding the C-terminal region (C-ter) of Nedd5, an intracytoplasmatic protein of the septin family. To evaluate anti-Nedd5 serum immunoreactivity, we used ELISA to measure specific IgG responses in patients with SLE classified according to the presence of psychiatric manifestations, such as psychosis and depression. Data were compared with those of patients with systemic sclerosis (an autoimmune disease characterized by endothelium damage and the presence of AECAs), of patients with infectious mononucleosis, and of healthy subjects. Finally, we investigated by immunofluorescence the intracellular redistribution of Nedd5 in endothelial cells after apoptotic stimuli.

## Materials and methods

### Patients

For the present investigation, we studied sera from an SLE cohort of 51 outpatients attending the Rheumatology Division of the University of Rome 'La Sapienza'. All patients were diagnosed according to the American College of Rheumatology revised criteria for the classification of SLE [2]. This population of SLE patients had been previously characterized with regard to their psychiatric and autoantibody profiles [5]. For our present purposes, we studied 50 of the 51 sera, because the serum from one SLE patient with mood disorder had run out. In this study, we also included the serum from a patient seen in the meantime with SLE and acute psychosis, a very rare manifestation of neuropsychiatric SLE.

Patients were categorized as being in group A or group B on the basis of the clinical psychiatric examination, which was performed by means of the Structured Clinical Interview for Psychiatric Diagnosis [12]. A psychiatric diagnosis was assigned according to the Diagnostic and Statistical Manual of Mental Disorders IV [13]. Group A consisted of patients with psychosis (*n *= 3) and mood disorders (*n *= 14). Group B included patients without psychiatric manifestations (*n *= 18) and patients whose only psychiatric manifestation was anxiety disorder (*n *= 16). We did not include patients with anxiety disturbance in group A, because in most SLE patients anxiety is considered a secondary stress reaction and not a direct manifestation of neuropsychiatric SLE [5].

Current SLE disease activity was measured using the SLE Disease Activity Index (SLEDAI). We also studied as controls sera from 35 sex- and age-matched healthy subjects; 20 sera from patients with systemic sclerosis (18 female, 2 male; mean age 53 years, range 27 to 72) attending the Rheumatology Division of the University of Rome 'La Sapienza'; and 20 sex- and age-matched patients with infectious mononucleosis from the Department of Experimental Medicine and Pathology of the University of Rome 'La Sapienza'. Informed consent was obtained from each patient, and the local ethics committee approved the study protocol. The sera were stored at -20°C until they were assayed.

### Immunoscreening of the cDNA expression library

A commercially available HUAEC cDNA library (Stratagene, Cambridge, UK) was used to screen for clones showing immunoreactivity with a serum from a patient with SLE, acute and active psychosis, and elevated titer of serum AECA. The expression library was screened essentially as previously described [14]. The serum was diluted 1:350 in PBS containing 1% milk and 0.05% Tween-20 and supplemented with 0.02% sodium azide. To reduce nonspecific binding to *Escherichia coli *(XL1-Blue MRF') (Stratagene) and phage vector, diluted pool was preadsorbed three times on nonrecombinant phage plaques. For primary immunoscreening, the library was plated out at 12,500 plaque-forming units per 140-mm plate, using XL1-Blue MRF' host cells in accordance with the supplier's instructions. In brief, nitrocellulose filters, incubated with 10 mM isopropyl β-D-1-thiogalactopyranoside (Sigma-Aldrich, St Louis, MO, USA) were overlaid onto the plates and incubated for 4 hours at 37°C. After blocking in 5% milk/PBS, the filters were incubated with the preadsorbed serum overnight at room temperature. After four washes with 0.05% PBS, Tween-20 membranes were incubated with a 1:3000 dilution of goat antihuman IgG (Bio-Rad, Richmond, CA, USA) in PBS containing 0.05% Tween-20 and 1% milk for 3 hours at room temperature. After a final four washes in 0.05% PBS Tween-20, membranes were incubated for 20 min with diaminobenzidine substrate (Sigma-Aldrich). Plaques corresponding to immunoreactive regions were cored from the original plate and resuspended in suspension medium containing 10 μl chloroform. Positive plaques were rescreened with the same serum to obtain the clonality.

Cloned phage showing immunoreactivity was recovered as pBluescript by single-stranded rescue using the helper phage (Stratagene) according to the supplier's instructions and used to transform SolR XL1cells. The nucleotide sequence of the cloned cDNA insertion was sequenced with automated sequencer ABI Prism 310 Collection (Applied Biosystems, Foster City, CA, USA) and sequences were then compared with the GenBank sequence database using both Fasta and Blast analysis [15,16]. To predict coiled-coil domain, we used the appropriate software .

### Expression and purification of the recombinant antigen

The selected cDNA clone was subcloned into the *Bam *HI/*Hind*III restriction site of the QIA express vector, pQE30. To obtain the whole molecule of Nedd5, we amplified the cDNA of the library using specific primers designed from the 5' and 3' termini of sequence obtained in GenBank (accession number BC033559) with *Bam *HI/*Hind*III restriction site and cloned in the expression vector.

The fusion protein was expressed in *Escherichia coli *SG130009 cells, purified by affinity of NI-NTA resin for the 6X histidine tag and eluted under denaturing conditions (urea) in accordance with the supplier's instructions (Qiagen, Hilden, Germany). After purification, urea was removed by dialysis in PBS with decreasing concentrations of urea, with a last change of PBS alone overnight at 4°C. Protein concentration was determined by the Bio-Rad Bradford protein assay (Bio-Rad).

### Indirect immunofluorescence assay

Hep-2 cells were directly stained with the mouse anti-Nedd5 polyclonal antiserum, obtained by standard immunization protocol, or with the corresponding mouse preimmune serum, in PBS containing 1% BSA. After washing three times with PBS, fluorescein-isothiocyanate (FITC)-conjugated antimouse IgG (γ-chain specific) (Sigma) were then added and incubation was at 4°C for 30 min.

Alternatively, EAhy926 human vascular endothelial cells [17] were grown to 60 to 70% confluence and seeded at 5 × 10^6 ^per well on glass cover slips. Cells, either untreated or treated with 20 ng/ml of tumor necrosis factor α (TNF-α) and 10 μg/ml of cycloheximide for 16 hours [18], were fixed with 4% formaldehyde in PBS for 30 min at 4°C. Alternatively, cells were permeabilized with acetone:methanol 1:1 (vol:vol) for 10 min at 4°C and then soaked in balanced salt solution (Sigma) for 30 min at 25°C. Cells were then incubated for 30 min at 25°C in the blocking buffer (2% bovine serum albumin in PBS, containing 5% glycerol and 0.2% Tween-20). Apoptosis was evaluated by propidium iodide staining, according to the method of Nicoletti and colleagues [19], and by the binding of FITC-conjugated Annexin V, using the Apoptest binding kit, containing annexin V-FITC and binding buffer. After washing three times with PBS, cells were incubated for 1 hour at 4°C with the mouse anti-Nedd5 polyclonal antiserum, obtained by standard immunization protocol, or with the corresponding mouse preimmune serum, in PBS containing 1% BSA. FITC-conjugated antimouse IgG (γ-chain specific) (Sigma) were then added and incubated at 4°C for 30 min. After washing with PBS, fluorescence was analyzed with an Olympus U RFL microscope (Olympus, Hamburg, Germany).

### SDS–PAGE and immunoblotting

Immunoblotting, after 12% SDS–PAGE under reducing conditions, was performed as previously described [20]. In brief, Nedd5 C-ter was used as antigen at the concentration of 3 μg/lane and was revealed by human sera diluted 1:100, by a monoclonal antibody specific to six-histidine tail (Qiagen), or by the mouse polyclonal antiserum (1:200). Goat antihuman and antimouse IgG-labelled sera (Bio-Rad) were used as second antibodies. Strips were developed with peroxidase substrate, 3-3' -diaminobenzidine (Sigma). EAhy926 endothelial cells were harvested by mechanical scraping and centrifuged at 10,000 ***g ***for 30 min and the pellet was resuspended in the loading buffer under reducing conditions, boiled for 10 min, and loaded (100,000 cells/well) in a 10.5% SDS-polyacrylamide gel. After immunoblotting, the mouse polyclonal antiserum and the corresponding mouse preimmune serum were used to reveal the presence of Nedd5 in the cell preparation.

### ELISA

ELISA for specific total IgG was developed essentially as previously described [14]. In brief, polystyrene plates (Dynex, Berlin, Germany) were coated with Nedd5 C-ter 0.5 μg/well in 0.05 μM NaHCO_3 _buffer, pH 9.5. Coated plates were incubated overnight at 4°C and then washed three times with PBS containing 0.05% Tween-20 in an automated washer (Wellwash 4, Labsystem, Turku, Finland). Plates were blocked with PBS Tween containing 3% gelatin (Bio-Rad), 100 μl/well, for 1 hour at room temperature and washed as previously described. Human sera were diluted in PBS Tween-20 and 1% gelatin (1:100 for total IgG) and pipetted onto plates at 100 μl per well. Plates were incubated for 1 hour at 20°C and washed as described. Peroxidase-conjugated goat antihuman IgG (Bio-Rad) was diluted 1:3000 in the same buffer. These dilutions were used as second antibodies and incubated (100 μl/well) for 1 hour at 20°C. *o*-Phenylenediamine dihydrochloride (Sigma) was used as a substrate and absorbance was measured at 490 nm. Means + 2 standard deviations (SD) of the absorbance reading of the healthy controls were considered the cutoff levels for positive reactions. All assays were performed in quadruplicate. Data were presented as the mean optical density (OD) corrected for background (wells without coated antigen). The results of unknown samples on the plate were accepted if internal controls (two serum samples, one positive and one negative) had an absorbance reading within mean ± 10% of previous readings. To inhibit specific IgG, the sera from three patients with SLE, anti-Nedd5 C-ter IgG positive, were diluted 1:50 in PBS-Tween and were incubated overnight at room temperature in 10 μg/ml of Nedd5 C-ter according to the method reported by Huang and colleagues [21]. As a negative control, the sera were pre-incubated with 40 μg/ml of BSA.

Cultures of human umbilical-vein-derived endothelial cells at the third to fourth passage were used to detect AECA (IgG), using a cell-surface ELISA on living cells, as previously reported [5]. AECAs were expressed as binding index (BI) equal to 100 × (S-A) / (B-A), where S is the OD of the sample tested, A is the OD obtained with only the secondary antibody, and B the OD of a positive reference serum. AECAs were considered positive when BI was higher than the cutoff value (mean+2 SD of 66 healthy controls) corresponding to 50% of a positive reference serum from a patients with SLE. Antibodies against cardiolipin, β2 glycoprotein I, Ro/SSA, Ro/SSA 52, La/SSB, glial fibrillary acidic protein, ribosomal P protein, and nucleosome IgG were tested as previously described [5]

### Statistical analysis

Unless otherwise specified, all values are means ± SD. The Fisher exact test and χ^2 ^analysis were used to evaluate differences between percentages; Student's *t*-test was used to evaluate differences between arithmetic means. *P *values less than 0.05 indicated statistically significant differences.

## Results

### Immunoscreening of HUAEC expression library

**Figure 1:**
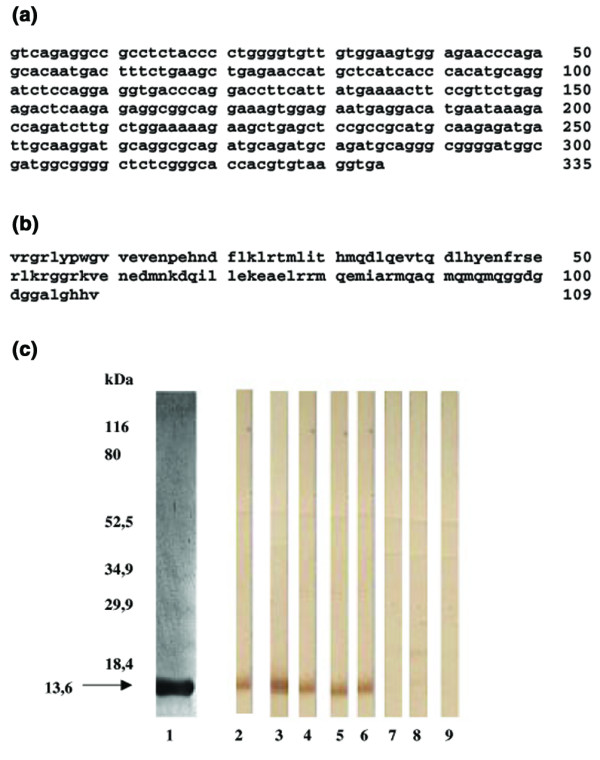
Nucleotide and amino acid sequences and immunochemical characterization of the C-terminal region of Nedd5. **(a) **The nucleotide sequence of 335 base pairs of the cloned cDNA insertion was sequenced with automated sequencer ABI Prism 310 Collection. **(b) **The amino acid sequence predicted from the nucleotide sequence is 109 residues long. The sequence compared with the GenBank sequence database using both Fasta and Blast analysis has 100% identity with the C-terminal subunit of Nedd5 (accession number Q15019). **(c) **The molecular size and the purity of the expressed protein were confirmed by 12% SDS–PAGE stained by Coomassie blue (lane 1). Immunoreactivity was analyzed by immunoblotting: monoclonal antibody antihistidine tail (lane 2); mouse polyclonal antiserum specific to Nedd5 C-ter (lane 3); representative sera from three patients with SLE IgG positive to Nedd5 (lanes 4,5,6); representative serum from a patient with SLE IgG negative to Nedd5 C-ter (lane 7); representative serum from a healthy subject (lane 8); control lane without serum (lane 9).

Immunoscreening of the HUAEC expression library with IgG from the serum of a patient with SLE and active psychosis identified a strongly reactive clone. The amino acid sequence, predicted from the 335-base-pair open reading frame of this clone, is 109 residues long and has 100% identity with the C-terminal subunit of Nedd5, a protein of the septin family (Fig. 1a,b). The search for possible coiled-coil domains in the database disclosed a coiled-coil domain in this amino acid region. Because preliminary experiments showed that the whole molecule and the C-terminal subunit gave equivalent serological results (data not shown), in serological tests we used the C-terminal subunit (Nedd5 C-ter), which contains the immunoreactive epitopes. The molecular size predicted from the amino acid sequence of 13.6 kDa, the purity, and the immunoreactivity of the expressed recombinant protein was confirmed by 12% SDS–PAGE and immunoblotting (Fig. 1c).

### ELISA for anti-Nedd5 C-ter IgG

IgGs specific to Nedd5 C-ter in ELISA were present in 14 (27.4%) of 51 SLE patients and did not correlate with the presence of AECAs previously studied in the same population of patients [5]. Moreover, no significant correlation was found between the presence of anti-Nedd5-C-ter IgG and the presence of antibodies against cardiolipin, β_2 _glycoprotein I, Ro/SSA, Ro/SSA52, La/SSB, glial fibrillary acidic protein, ribosomal P protein, or nucleosome IgG (data not shown). To assess the specificity of ELISA, we preadsorbed sera from three SLE patients positive to Nedd5 C-ter with Nedd5 C-ter itself, and we observed a complete inhibition of reactivity (data not shown).

**Figure 2:**
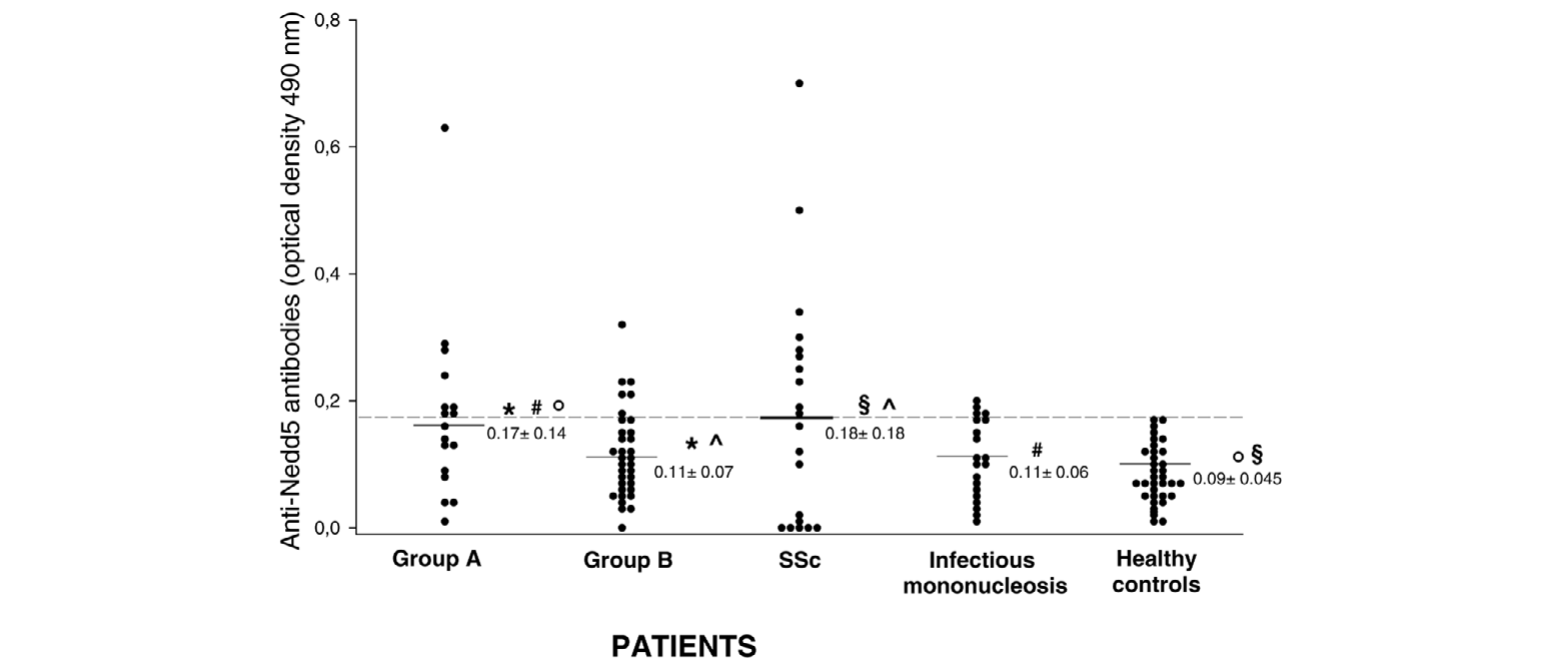
Anti- Nedd5 C-ter antibodies in patients with SLE with and without psychiatric manifestations. Dot plot of anti-Nedd5 C-ter IgG in systemic lupus erythematosus (SLE) patients with psychiatric manifestations (group A, *n *= 17), in SLE patients without psychiatric manifestations other than anxiety (group B, *n *= 34), in systemic sclerosis (SSc) patients (*n *= 20), and in patients with infectious mononucleosis (*n *= 20). Each dot represents a subject. The samples were considered positive when their optical density was higher than the cutoff value (mean + 2 SD for 35 healthy controls). The broken line represents the cutoff (0.18). *Group A vs group B, *P *= 0.04; #group A vs infectious mononucleosis, *P *= 0.034; °group A vs healthy controls, *P *= 0.003; ^SSc patients vs group B, *P *= 0.046; §SSc patients vs healthy controls, *P *= 0.003 (Student's *t*-test).

The mean OD value for IgG immunoreactivity to Nedd5 C-ter was significantly higher in patients of group A than in those of group B, those with infectious mononucleosis, or healthy subjects (0.17 ± 0.14 vs, respectively, 0.11 ± 0.07, *P *= 0.04; 0.11 ± 0.06, *P *= 0.034; 0.09 ± 0.045, *P *= 0.003, on Student's *t*-test). Moreover, IgG immunoreactivity to Nedd5 C-ter was significantly higher in patients with systemic sclerosis than in patients of group B or in healthy subjects (0.18 ± 0.18 vs, respectively, 0.11 ± 0.07, *P *= 0.046; and 0.09 ± 0.045, *P *= 0.003) (Fig. 2). The percentage of patients with anti-Nedd5 C-ter serum IgG was higher in group A than in group B (8 (47%) of 17, vs 6 (17%) of 34, *P *= 0.045 on the Fisher exact test). No correlation was observed between the presence of anti-Nedd5 C-ter antibodies and clinical features of SLE or SLEDAI (Table 1).

### Localization of Nedd5 in HEp-2 cells and in EAhy926 cells

**Figure 3:**
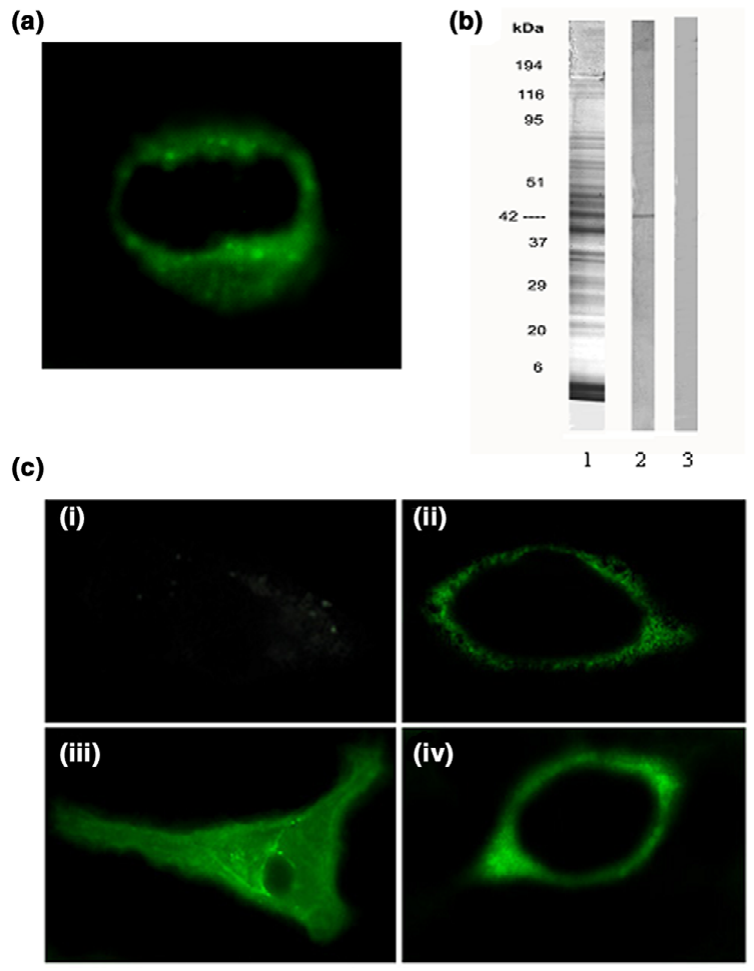
Localization of Nedd5 in HEp-2 cells and in EAhy926 cells. **(a) **Immunofluorescence analysis of Nedd5 localization in HEp-2 cells. The mouse polyclonal antiserum specific to Nedd5 C-ter was used to analyze the cellular distribution of Nedd5. **(b) **10.5% SDS–PAGE and immunoblotting of EAhy926 cells. EAhy926 cells were centrifuged and the pellets were redissolved in the loading buffer under reducing conditions (100,000 cells/well). 10.5% SDS–PAGE stained by Coomassie blue (1); immunoblotting was performed with the mouse polyclonal antiserum specific to Nedd5 C-ter (2) and with the mouse preimmune serum (3). **(c) **Immunofluorescence analysis of Nedd5 localization in endothelial cells under physiological conditions and during apoptosis. EAhy926 cells were treated with tumor necrosis factor α (TNF-α) (20 μg/ml) plus cycloheximide (10 μg/ml) for 16 hours to induce apoptosis. The mouse polyclonal antiserum specific to Nedd5 C-ter was used to analyze the cellular distribution of Nedd5. **(i) **Untreated cells, fixed with 4% formaldehyde in PBS; **(ii) **TNF-α plus cycloheximide-treated cells, fixed with 4% formaldehyde in PBS; **(iii) **untreated cells, permeabilized with acetone:methanol 1:1 (vol:vol); **(iv) **TNF-α plus cycloheximide-treated cells, permeabilized with acetone : methanol 1:1 (vol:vol).

We analyzed primarily the localization of Nedd5 in HEp-2 cells, a conventional cell line used in clinical laboratories because of their active proliferation. As expected, the immunolabelling with a mouse polyclonal antiserum specific to the recombinant Nedd5 C-ter appeared confined to the cytoplasm, where staining of the contractile ring was evident. No staining was observed on the cell surface (Fig. 3a). In contrast, no staining with the mouse preimmune serum was observed, indicating that the immunolabelling was specific for Nedd5 C-ter (data not shown).

Immunoblotting of EAhy926 cells, revealed with the mouse polyclonal antiserum specific to the recombinant Nedd5 C-ter, showed a single band corresponding to the native Nedd5 protein at the expected molecular size of 42 kDa deduced from the amino acid sequence (Fig. 3b).

We analyzed Nedd5 relocation to the plasma membrane of EAhy926 cells during apoptosis, by immunofluorescence assay with the mouse polyclonal antiserum anti-Nedd5 C-ter (Fig. 3c). Untreated cells showed virtually no staining on the plasma membrane (i). On the contrary, an uneven surface distribution of anti-Nedd5 staining was observed in cells treated with TNF-α plus cycloheximide (ii). These findings were confirmed in permeabilized cells. Indeed, untreated control cells showed anti-Nedd5 staining confined to the cytoplasm, with pronounced perinuclear granularity and without significant staining of the cell surface (iii). Induction of apoptosis by treatment with TNF-α plus cycloheximide changed the cellular distribution of Nedd5, with an uneven surface distribution of the staining that appeared in the cytoplasm and around plasma membrane (iv). No staining with the mouse preimmune serum was observed in any of the samples, indicating that the observed immunolabelling was specific for Nedd5. Apoptosis was checked by testing the exposure of phosphatidylserine on the cell surface by the binding of FITC-conjugated Annexin V, which revealed that up to 80% of the cells were positive (data not shown).

## Discussion

In this study, we used a molecular cloning strategy to identify endothelial autoantigens in SLE patients. Results provide evidence that the C-terminal region of Nedd5 is a novel autoantigen with a role in neuropsychiatric manifestations. Nedd5 is a mammalian septin known to associate with actin-based structures such as the contractile ring and stress fibers [22,23]. The septins are a family of cytoskeletal GTPases that play an essential role in cytokinesis in yeast and mammalian cells [24]. Nedd5 is predominantly expressed in the nervous system and may contribute to the formation of neurofibrillary tangles as integral constituents of paired helical filaments in Alzheimer's disease [25,26].

To our knowledge, this is the first report describing an immune response against a protein of the septin family. This study provides evidence that Nedd5 molecules are expressed on the cell surface after apoptotic stimuli, suggesting a possible mechanism by which Nedd5 may be autoantigenic. Indeed, apoptosis may play an important role in bypassing tolerance to intracellular autoantigens. The specific modification of autoantigens and their redistribution into blebs at the surface of apoptotic cells may contribute to the induction of autoimmune responses [27,28]. Moreover, apoptotic defects and impaired removal of apoptotic cells could contribute to an overload of autoantigens in the circulation or in target tissues that could become available to initiate an autoimmune response [29]. In susceptible individuals, this can lead to autoantibody-mediated tissue damage.

Interestingly, the C-terminal region of Nedd-5 displays a coiled-coil domain. Several autoimmune autoantigens are characterized by the presence of such a domain [30]. Coiled-coil proteins may be exposed to the immune system as surface structures in aberrant disease states associated with unregulated cell death and could become autoimmune targets [30].

Even though in this study we used an endothelial cDNA expression library and we screened it with a serum with an elevated AECA titer, we found no significant correlation between the presence of AECAs and the presence of anti-Nedd5 antibodies in patients with SLE (data not shown). This finding is not surprising, since the cell-surface ELISA on living cells used to detect AECAs reveals only plasma membrane antigens, whereas Nedd5, which is normally confined within the cytoplasm, becomes exposed on the cell surface after triggering apoptosis. Interestingly, we found such antibodies in a large proportion of patients with systemic sclerosis, a pathology in which endothelial damage may often occur. However, we cannot exclude the possibility that the autoimmune response we observed was generated against Nedd5 present in other cellular compartments, such as the nervous system.

An association between serum AECAs and psychosis or depression in patients with SLE has been recently reported, strengthening the view of a possible implication of AECAs in the development of psychiatric disorders in SLE [5]. In this study, attempting to identify a possible molecular target of AECAs in an SLE patient with active psychosis, analyzing the same population of patients as in the previous investigation [5], we demonstrated an association between serum IgG specific to the C-terminal region of Nedd5 and psychiatric manifestations in patients with SLE. Notably, all of the three patients with psychosis had serum IgG to Nedd5 C-ter. Overall, although anti-Nedd5 autoantibodies are not specific to SLE, they are significantly associated with neuropsychiatric SLE and could be immunological markers of psychiatric manifestations in this pathology. The unanswered question is whether anti-Nedd5 C-ter antibodies can cause direct damage, thus contributing to the pathogenesis of psychiatric manifestations, or whether they are an epiphenomenon of these disorders. Further studies are in progress in order to clarify the effective role of anti-Nedd5 C-ter antibodies *in vivo*.

## Conclusion

In the present study, we identified Nedd5 C-ter as a novel autoantigen in SLE. This result is of clinical importance and may be a valuable tool in the diagnosis of neuropsychiatric SLE. In addition, having this recombinant antigen may help in defining the precise role that specific autoantibodies may play in the autoimmune mechanisms underlying psychiatric manifestations in SLE.

## Abbreviations

AECA = anti-endothelial-cell antibody; BSA = bovine serum albumin; C-ter = C-terminal region; ELISA = enzyme-linked immunosorbent assay; FITC = fluorescein isothiocyanate; HUAEC = human umbilical artery endothelial cell; OD = optical density; PBS = phosphate-buffered saline; SD = standard deviation; SLE = systemic lupus erythematosus; SLEDAI = SLE Disease Activity Index; TNF-α = tumor necrosis factor α.

## Competing interests

The author(s) declare that they have no competing interests

## Authors' contributions

PM carried out the screening of the library and participated in the design of the study and in the analysis of data. MS carried out the experiments on endothelial cell line and apoptosis and participated in the design of the study and helped to draft the manuscript. FC participated in the design of the study and in analysis of data and helped to draft the manuscript. FD carried out the cloning and sequencing of cDNA and protein expression and contributed in the interpretation of data. MR carried out the ELISA experiments and participated in analysis of data. CA performed the statistical analysis and the clinical associations. AS participated in the analysis and interpretation of data and in the revision of the manuscript. RR participated in the design of the study and in the revision of the manuscript. EP participated in analysis of data. GV participated in the design and revision of the study. EO conceived of the study, participated in its design and coordination, and drafted the manuscript. All authors read and approved the final manuscript.

## Figures and Tables

**Table 1:** Clinical characteristics of SLE patients according to psychiatric symptoms and anti-Nedd5 C-ter IgG

	Group A^a ^(*n *= 17)		Group B^a ^(*n *= 34)	
Characteristic	Anti-Nedd5 C-ter IgG (*n *= 8)	No anti-Nedd5 C-ter IgG (*n *= 9)	Anti-Nedd5 C-ter IgG (*n *= 6)	No anti-Nedd5 C-ter IgG (*n *= 28)

Age (y), mean (range)	38.6 (26–52)	37.1 (23–50)	34.5 (28–48)	37.2 (14–70)
Sex (males/females)	1/7	2/7	1/5	3/25
Disease duration (y), mean (range)	11.2 (7–21)	6.8 (0.5–19)	9 (5–15)	12.1 (0.5–24)
Arthritis, no. (%)	5 (62.5)	9 (100)	5 (83.3)	18 (64.2)
Neurological involvement, no. (%)	2 (25)	4 (44.4)	2 (33.3)	11 (39.2)
Renal involvement, no. (%)	3 (37.5)	0	3 (50)	9 (32.1)
Cytopenia, no. (%)	5 (62.5)	7 (77.7)	4 (66.6)	16 (57.1)
Serositis, no. (%)	2 (25)	2 (22.2)	2 (33.3)	8 (28.5)
SLEDAI >3, no. (%)	5 (62.5)	4 (44.4)	4 (66.6)	12 (42.8)

^a^Group A, patients with depression or psychosis; group B, patients without psychiatric manifestations other than anxiety. Differences between the groups as measured by the χ^2 ^test were not statistically significant. SLE, systemic lupus erythematosus; SLEDAI, Systemic Lupus Erythematosus Disease Activity Index.
